# Dynamical Modeling of the Core Gene Network Controlling Transition to Flowering in *Pisum sativum*

**DOI:** 10.3389/fgene.2021.614711

**Published:** 2021-03-11

**Authors:** Polina Pavlinova, Maria G. Samsonova, Vitaly V. Gursky

**Affiliations:** ^1^Mathematical Biology and Bioinformatics Laboratory, Peter the Great Saint Petersburg Polytechnic University, Saint Petersburg, Russia; ^2^Theoretical Department, Ioffe Institute, Saint Petersburg, Russia

**Keywords:** pea, gene network, flowering initiation, differential equations, neural networks, dynamical model

## Abstract

Transition to flowering is an important stage of plant development. Many regulatory modules that control floral transition are conservative across plants. This process is best studied for the model plant *Arabidopsis thaliana*. The homologues of *Arabidopsis* genes responsible for the flowering initiation in legumes have been identified, and available data on their expression provide a good basis for gene network modeling. In this study, we developed several dynamical models of a gene network controlling transition to flowering in pea (*Pisum sativum*) using two different approaches. We used differential equations for modeling a previously proposed gene regulation scheme of floral initiation in pea and tested possible alternative hypothesis about some regulations. As the second approach, we applied neural networks to infer interactions between genes in the network directly from gene expression data. All models were verified on previously published experimental data on the dynamic expression of the main genes in the wild type and in three mutant genotypes. Based on modeling results, we made conclusions about the functionality of the previously proposed interactions in the gene network and about the influence of different growing conditions on the network architecture. It was shown that regulation of the *PIM*, *FTa1*, and *FTc* genes in pea does not correspond to the previously proposed hypotheses. The modeling suggests that short- and long-day growing conditions are characterized by different gene network architectures. Overall, the results obtained can be used to plan new experiments and create more accurate models to study the flowering initiation in pea and, in a broader context, in legumes.

## Introduction

Flowering is associated with a significant physiological change in plant development which manifests the transition from vegetative growth to reproductive development. For the reproductive success of plants, it is important for this transition to occur at the most appropriate moment. Various exogenous and endogenous pathways contribute to the control for the flowering time, and these pathways are best studied for the model plant *Arabidopsis thaliana* (Srikanth and Schmid, 2011; Andrés and Coupland, 2012; Khan et al., 2014). The key factor in the activation of the photoperiodic pathway of flowering initiation in *Arabidopsis* is the protein encoded by the *FLOWERING LOCUS T* (*FT*) gene. The FT, a phosphatidylethanolamine binding protein (PEBP), is a mobile signal transported from the leaves to the top of the shoot apex, where it promotes the plant’s transition to flowering. Expression of the *FT* gene depends on the influence of external and internal signals, which allow the plant to regulate the flowering initiation time (Kardailsky et al., 1999; Kobayashi et al., 1999; Jaeger et al., 2013). After synthesis in the leaves, the FT protein moves to the shoot apical meristem and forms a complex with the bZIP-type transcription factor FLOWERING LOCUS D (FD; Abe et al., 2005), which belongs to the 14-3-3 protein family (Taoka et al., 2011). Main target genes of the FT-FD complex are the flower meristem identity gene *AP1* (*APETALA1*; Wigge et al., 2005) and *SUPPRESSOR OF OVEREXPRESSION OF CONSTANS1* (*SOC1*; Yoo et al., 2016). The latter is an activator of the gene *LEAFY* (*LFY*), which also controls the transition of shoot apical meristems to flower meristems (Lee et al., 2008). The flower meristem identity genes *AP1* and *LFY* transcriptionally activate each other (Jaeger et al., 2013).

**Figure 1:**
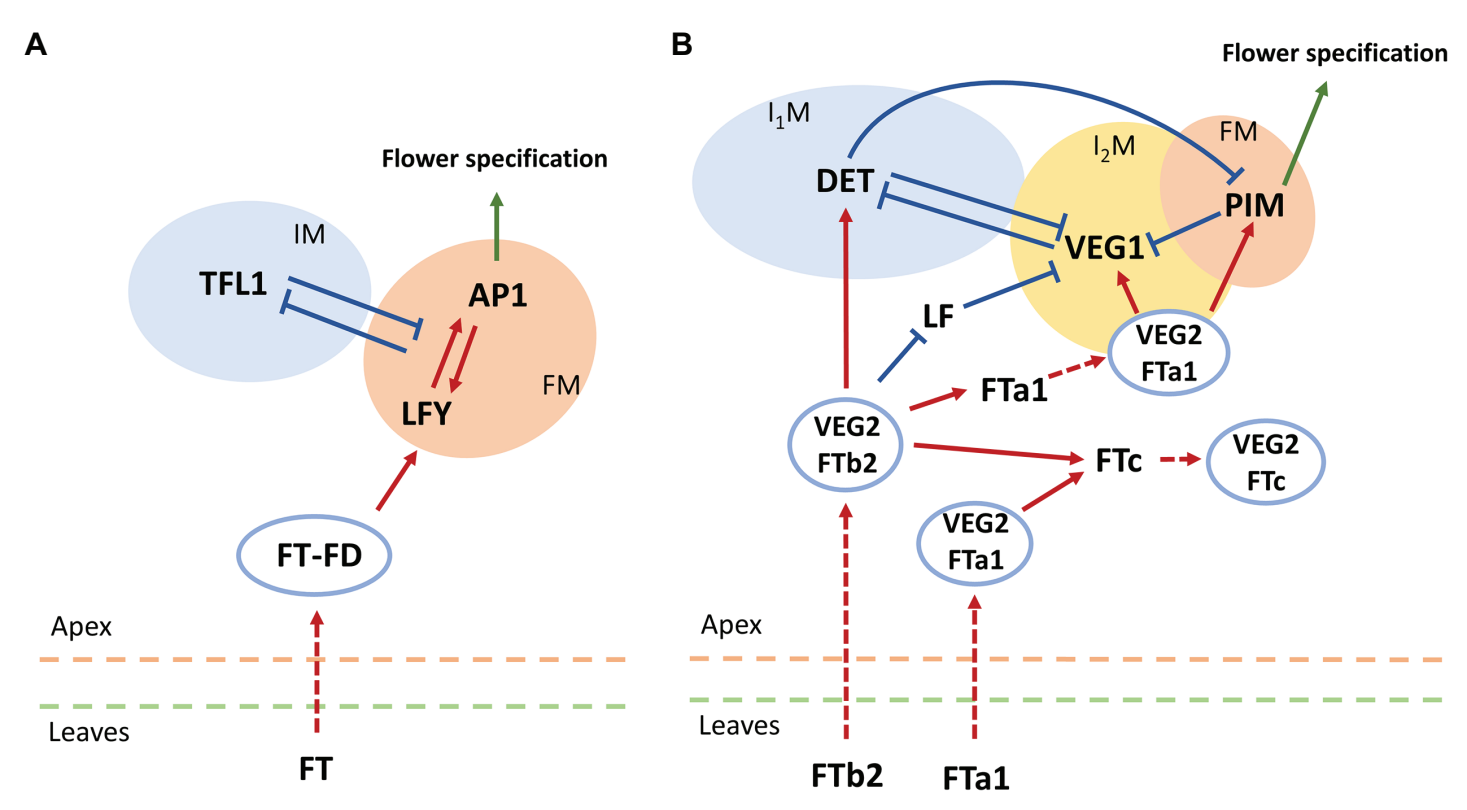
Schemes for genetic control of floral initiation in **(A)** *Arabidopsis* and **(B)** pea (*Pisum sativum*). **(A)** The core gene network controlling floral transition in *Arabidopsis thaliana* (Jaeger et al., 2013). IM, inflorescence meristem; FM, flower meristem. **(B)** The core gene network controlling floral transition in pea. FTb2 is expressed in plant leaves under LD conditions; it then moves to the apex, where it interacts with VEGETATIVE2 (VEG2). The resulting complex VEG2-FTb2 stimulates the formation of the primary inflorescence meristem (I_1_M) by activating the meristem identity gene *DETERMINATE* (*DET*). Within the apex, FTb2 also activates *FTa1* and *FTc* and downregulates *LF*. *FTa1* is expressed both in the leaves and in the apex. By means of the complex with VEG2, FTa1 probably stimulates *FTc* expression and activates *VEGETATIVE1* (*VEG1*). The latter activation leads to higher expression of *VEG1*, enhanced by the reduced repression from LF, and this expression initiates the secondary inflorescence meristem formation (I_2_M). FTa1-VEG2 also activates the floral meristem (FM) identity gene *PROLIFERATING INFLORESCENCE MERISTEM (PIM)*. The mutual repression between the three meristem identity genes (*DET*, *VEG1*, and *PIM*) ensures a spatial separation of the corresponding developmental compartments, maintaining the indeterminate inflorescence development. Dashed arrow-headed lines indicate movement of proteins from leaves to apex and protein complex formation within the apex. Red solid arrow-headed lines correspond to transcriptional activation, and blue solid T-like lines indicate transcriptional repression. The regulation scheme is based on a figure by Sussmilch et al., 2015.

The balance between activation and repression of flowering initiation is important for plants with indeterminate inflorescence architecture, in which newly forming flowers do not stop further plant growth (Benlloch et al., 2015). The key repressor of flowering initiation in *Arabidopsis* is the gene *TERMINAL FLOWER1* (*TFL1*), which is a close relative of *FT* and encodes a protein belonging to the PEBP family. This protein is expressed during floral transition in the center of the shoot apical meristem and maintains it in the vegetative state by suppressing the expression of *LFY* and *AP1* (Jaeger et al., 2013; Goretti et al., 2020). In turn, AP1 represses *TFL1* by directly binding its regulatory elements (Kaufmann et al., 2010). This mutual repression between *TFL1* and *LFY*/*AP1* explains the inflorescence meristem maintenance and flower meristem formation on its flanks (Benlloch et al., 2015). The minimal graph summarizing the genetic control of the photoperiod pathway in flower transition in *Arabidopsis* is shown in Figure 1A.

During evolution of legumes, the floral transition regulation has become more complex. This class of plants is characterized by the formation of a more complicated, the so-called compound, inflorescence architecture (Benlloch et al., 2015). In the process of growth, two meristems (primary and secondary) are successively formed. Moreover, multiple copies of the PEBP genes were identified in legumes homologous to *FT* and *TFL1*, associated with multiple genome duplication events during evolution (Hecht et al., 2011). The legume *FT*-like genes are subdivided into three subclasses: *FTa*, *FTb*, and *FTc*. Five *FT*-like genes from these subclasses were identified in pea (*Pisum sativum*; *FTa1*, *FTa2*, *FTb1*, *FTb2*, and *FTc*). These genes are characterized by variable expression patterns under different conditions. Under long day (LD) conditions, *FTa1* and *FTb2* are expressed in the leaves, while under short day (SD) conditions only decreased expression of *FTa1* is observed. In the plant apex, only *FTc* and *FTa1* are expressed. Such differences indicate distinct functions of the *FT* genes in floral initiation in pea (Hecht et al., 2011).

Pea homologues of the flower meristem identity genes *AP1* and *LFY* are *PROLIFERATING INFLORESCENCE MERISTEM* (*PIM/PEAM4*) and *UNIFOLIATA* (*UNI*), respectively (Hofer et al., 1997; Taylor et al., 2002). Homologues of the floral repressor *TFL1* in pea include *DETERMINATE* (*DET*), which is a marker of the primary inflorescence meristem (Berbel et al., 2012), and *LATE FLOWERING* (*LF*), whose function is not entirely clear (Foucher et al., 2003). The secondary inflorescence meristem is under control of *VEGETATIVE1* (*VEG1*; Berbel et al., 2012). A pea homologue of *FD* is *VEGETATIVE2* (*VEG2*), which is thought to form the complex with FTs similarly to *Arabidopsis* (Sussmilch et al., 2015).

Hecht et al. (2011) qualitatively analyzed the expression of these genes in pea, both in the leaves and in the shoot apical meristem, under different growth conditions and genotypes. Later, Sussmilch et al. (2015) proposed a scheme for regulations underlying the compound inflorescence development and floral transition in pea, as depicted in Figure 1B. In our study, we apply modeling to test whether the proposed regulation scheme fits the expression data quantitatively.

Methods of mathematical modeling are widely applied to the analysis of gene networks. These methods include Boolean models, ordinary differential equations (ODEs), neural networks, Bayesian networks, and stochastic modeling (Chai et al., 2014; Le Novère, 2015). The choice between different modeling approaches depends on the type of data used to calibrate the model.

Various modeling techniques were used for the quantitative analysis of gene networks involved in plant growth and development (Haque et al., 2019), in particular, in the photoperiodic pathway of floral transition. A method of neural networks was applied to study the transition to flowering of *Arabidopsis* (Welch et al., 2003). This model had a prescribed neural network architecture and described the interaction of the main genes responsible for various pathways of flowering initiation in the plant. The model was trained on values of such phenotypic parameters as the daylight length and the number of days after sowing. Later, the main regulatory elements underlying the photoperiodic pathway of *Arabidopsis* transition to flowering were identified using a dynamical model based on differential equations, which was applied to the data on flowering time of the wild and mutant genotypes (Jaeger et al., 2013). It was shown that the dynamics of flowering initiation can be explained by dividing the gene network into several feedback and forward loops with specific functional roles (Pullen et al., 2013). A more advanced model was developed later by Leal Valentim et al. (2015), in which additional regulators (SOC1 and AGL24) were added into the activation of *LFY* by the FT-FD complex, and the model was fitted to gene expression data. This approach allowed to test various hypotheses about *LFY* regulation by SOC1 and AGL24 and elucidated a nonlinear nature of the flowering network. Wang et al. (2014) investigated different approximations used to formulate model equations and compared their influence on the model performance in describing floral initiation in *Arabidopsis*. Apart from *Arabidopsis*, similar models of floral transition were also elaborated for chickpea (*Cicer arietinum*), which is a member of the legume family. Like pea, it has multiple homologous of the *FT* and *TFL1* genes (Ridge et al., 2017). A dynamical model of the flowering gene network was developed and used for testing various hypotheses on how the *FT*- and *TFL1*-like genes combine in regulating the flower meristem identity genes in the ICCV 96029 chickpea cultivar (Gursky et al., 2018). The same model was not successful for CDC Frontier, which is another chickpea cultivar. A machine learning-based modeling approach was developed and applied for this cultivar, predicting that SD and LD growing conditions may be associated with different architectures of the flowering gene network (Podolny et al., 2020). Extending a classical qualitative model for the control of flowering initiation, Wenden et al. (2009) elaborated a quantitative model of flowering in pea (Wenden and Rameau, 2009). This model was used to formulate new hypotheses about the signals controlling flowering. More sophisticated modeling and software platforms were proposed taking into account mechanical processes during flower development and, more generally, morphogenesis in plants, and using advanced data quantification methods (Barbier de Reuille et al., 2015; Boudon et al., 2015).

We extend the previous modeling attempts to floral transition in pea. We construct several dynamical models and apply them to the previously published data on the photoperiodic pathway of flowering initiation in pea (Hecht et al., 2011; Sussmilch et al., 2015). We specifically investigate the compatibility of the network from Figure 1B to the data at the quantitative level.

## Results

We calibrated our models on the previously published dynamic expression data of genes responsible for flowering initiation in pea (cultivar NGB5839; Hecht et al., 2011; Sussmilch et al., 2015). We extracted the expression data for three *FT*-like genes (*FTa1*, *FTb2*, and *FTc*), two homologues of the *TFL1* gene (*DET* and *LF*), one homologue of the *FD* gene (*VEG2*), a homologue of the flower meristem identity gene *AP1* (*PIM*), and the *VEG1* gene responsible for secondary meristem formation. For all genes except *VEG1*, data were available for the SD and LD growth conditions in the wild type; *VEG1* expression data were available only for LD. In addition, expression data for the same genes were extracted for three mutant genotypes: *late1-2*, *dne-1*, and *gigas-2*. *late1-2* is a mutant for gene *LATE1*, which has delayed flowering under LD. *dne-1* represents a mutant for gene *DNE1*, which starts flowering under SD at the same time as a wild-type plant under LD. *gigas-2* is the *FTa1* null mutant.

### Dynamical Models Based on the Proposed Regulation Scheme

**Figure 2:**
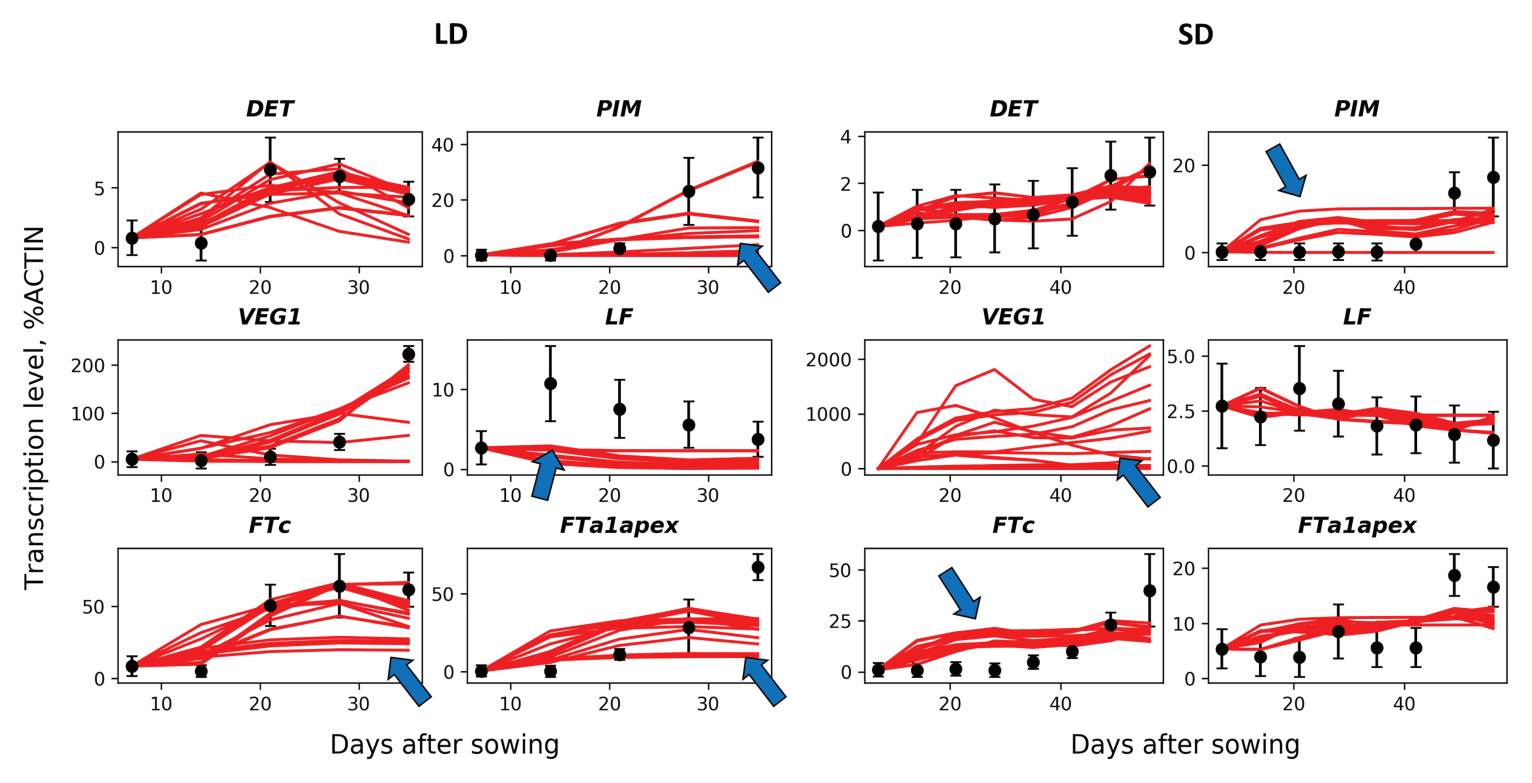
Solutions in the baseline MM model in comparison with the wild-type data. The model solutions (red curves) corresponding to all parameter sets found by multiple optimization runs are shown for six genes and for the short day (SD, right panels) and long day (LD, left panels) conditions. The black dots and error ranges are the mean expression data and standard deviations, respectively, extracted from (Hecht et al., 2011; Sussmilch et al., 2015). The arrows indicate the most significant discrepancies between the solutions and data.

We developed a dynamical model describing gene expression according to the regulation scheme shown in Figure 1B. We formulated ODEs implementing the Michaelis–Menten kinetics for the expression of each gene under the influence of its regulators and fitted this model to the expression data, in order to understand how the proposed regulation scheme matches the data at the quantitative level. We first investigated a baseline model [the MM model; equations (1)–(11) in Materials and Methods] which includes only regulations shown in Figure 1B and, in particular, considers the competitive binding of VEG2 by FTa1, FTb2, and FTc. We found values of free parameters by fitting this model to the wild-type expression data. In order to reduce the probability of overfitting, we analyzed all solutions resulted from a series of the numerical optimization runs (Figure 2). These solutions qualitatively match the data dynamics but have several quantitative discrepancies. In SD, insufficient repression at early times and insufficient activation at later times of *PIM* and *FTc* are observed. As data for VEG1 were absent in SD, the solution for this protein was not fitted to data. As a consequence, most of the VEG1 solutions have unrealistically high expression values in SD. The defects in LD include deficient activation at later times in most solutions for PIM, FTc, and apical FTa1, and deficient activation of *LF* at early times. Testing the model on the data from mutants also showed a qualitative correspondence between the model and the data, but with quantitative defects (Supplementary Figures 1, 2).

### Testing Alternative Hypotheses About Gene Regulations

**Figure 3:**
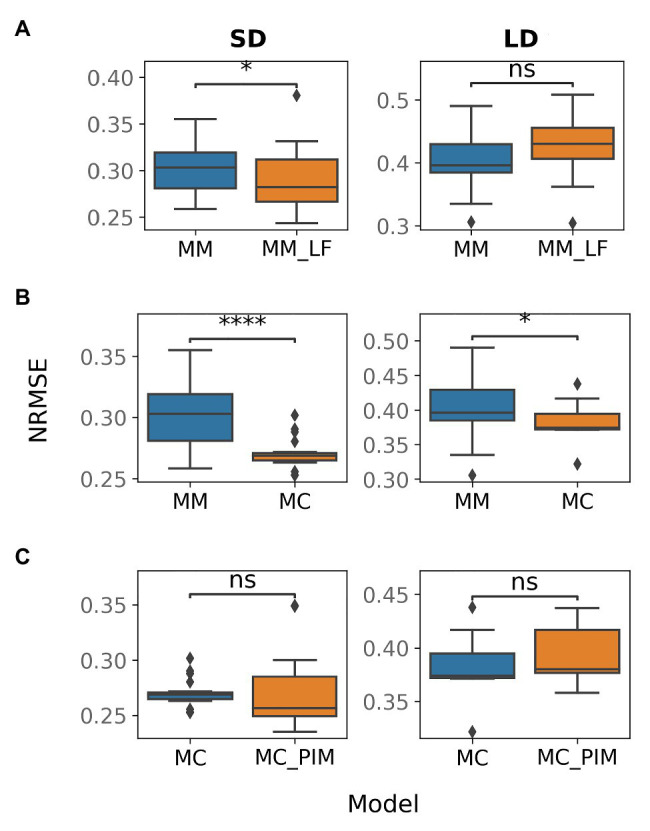
Normalized root-mean-square error (NRMSE) calculated on the wild-type data and solutions obtained from multiple optimization runs in various models. NRMSE is shown for the following couples of a baseline and alternative models: **(A)** MM and MM_LF, **(B)** MM and MC, **(C)** MC and MC_PIM. The model names are introduced in the text. The Mann–Whitney–Wilcoxon test was applied to check that the alternative model provides better performance (smaller NRMSE) than the baseline model; *p*-values: (ns) 0.05 < *p* ≤ 1, (^*^) 0.01 < *p* ≤ 0.05, (^****^) *p* ≤ 10−4.

To improve the baseline model, we tested several alternative hypotheses about additional interactions in the gene network. TFL1 inhibits floral initiation in *Arabidopsis* by repressing expression of *AP1*. Among two pea homologues of the *TFL1* gene (*DET* and *LF*), only *DET* was suggested as a repressor of *PIM*, which is the pea homologue of *AP1* (Figure 1B). We assumed that *LF* also represses *PIM* and that this repression would reduce overexpressed *PIM* at early times in SD. To test this hypothesis, we formulated the MM_LF model by adding the new regulation into equations of the baseline MM model [see equation (12) in Materials and Methods] and fitted the new model to the wild-type data. The MM_LF model showed a slightly better performance as compared to the MM model in SD, but the performance became worse in LD (Figure 3A). Taking into account that the early dynamics of PIM is not improved essentially (Supplementary Figure 3) and MM_LF is much worse than MM on data from the *gigas-2* mutant (Supplementary Figure 4), we can reject the hypothesis about *PIM* repression by LF.

On the next step, we tested whether the competitive binding of VEG2 by FTa1, FTb2, and FTc is essential. We adjusted the MM model by assuming that the binding is uncompetitive, so that the concentrations of the complexes were taken equal to the product of the concentrations of the corresponding proteins [equations (13)–(15) in Materials and Methods]. This new alternative model (MC model) demonstrated a better performance on the wild-type data as compared to the MM model, both in SD and in LD (Figure 3B), and was also better than the MM model on the data from mutant genotypes (Supplementary Figure 4; Supplementary Table 3). The wild-type solutions in the MC model are less variable than in the MM model and show improvement in expression dynamics of *PIM* and *FTc* in LD (Supplementary Figure 5). VEG1 in the MC model also has a more reasonable expression dynamics range in SD (Supplementary Figure 5). These results suggest that the binding of VEG2 by the FT proteins is uncompetitive.

In SD, the MM and MC models both have solutions with an overstated early and understated late expression of *PIM*. One possible solution to this problem is to make the dynamical curve of PIM respond in a more nonlinear way to the monotonically increasing expression curve of the complex VEG2-FTa1, which is the only activator of *PIM*. This nonlinearity can be achieved by adding a cooperativity parameter into the model, responsible for the putative cooperative binding of VEG2-FTa1 to the *PIM* promoter. It was shown that homologues of the FD (VEG2) and FT proteins form a complex consisting of several subunits in rice (*Oryza sativa*), thus sustaining the hypothesis about cooperative regulation by the VEG2-FT complexes (Taoka et al., 2011; Tsuji et al., 2013). We implemented the cooperativity hypothesis into the MC model by assuming that the cooperativity parameter *n* (*n* > 1) in the term responsible for the regulation of *PIM* by VEG2-FTa1 is an additional free parameter (MC_PIM model). However, the new model neither improved the total performance as compared to the MC model (Figure 3C) nor fixed the *PIM* expression dynamics in SD (Supplementary Figure 6), thus suggesting that the regulation of *PIM* is noncooperative.

### Models Trained on Full Data

The models described above were trained on the wild-type data and tested on the mutant data. In these computational experiments, the MC model outperformed other alternative models on both the wild-type and mutant data (Figure 3; Supplementary Figure 4). However, most of the defects shown for the baseline MM model persisted in the MC model. In order to increase the amount of data used to optimize parameter values, we used the same equations as in the MC model and fitted them to gene expression data for all genotypes (wild type, *dne-1*, *late1-2*, and *gigas-2*). Since we used all the available data to fit the model, we aimed to investigate the maximal possible performance of the model in this computational experiment. Later, we will split both the wild-type and mutant data into training and testing subsets when modeling with neural networks.

We refer to this model trained on the complete data set as MC_Cdata. In terms of the normalized error, the MC_Cdata model expectedly outperforms MC on the mutant data but has a bit worse performance on the wild-type data (Supplementary Figure 7). However, the wild-type expression dynamics is qualitatively similar in the two models (Supplementary Figures 5, 8).

**Figure 4:**
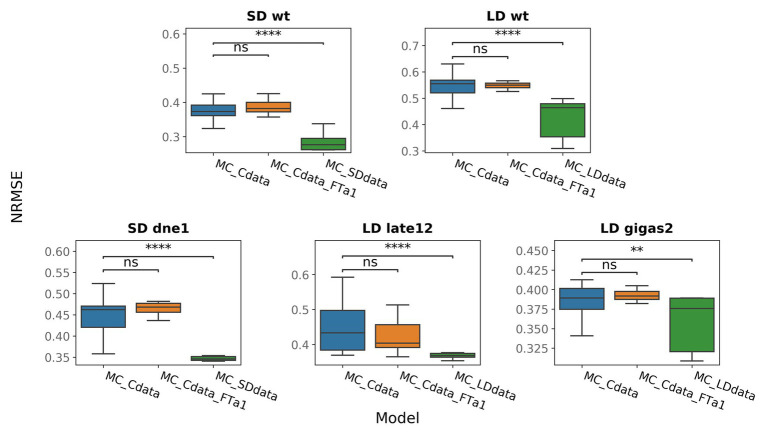
NRMSE in models trained on the full data (MC_Cdata and MC_Cdata_FTa1) or on the SD and LD portions of the full data (MC_SDdata and MC_LDdata, respectively). The Mann–Whitney–Wilcoxon test was applied to check that the alternative models provide better performance (smaller NRMSE) than the MC_Cdata model; *p*-values: (ns) 0.05 < *p* ≤ 1, (^**^) 0.001 < *p* ≤ 0.01, (^****^) *p* ≤ 10^−4^.

One of the defects observed in all models is a low FTa1 concentration at later times in the wild type (Figure 2; Supplementary Figures 5, 8). According to the proposed regulation scheme, *FTa1* in the apical meristem is activated only by the VEG2-FTb2 complex. In order to add activation to the *FTa1* expression, we suggested that FTa1 activates its own production in the apex. We tested this hypothesis by inserting an additional term into the equation for FTa1 that characterized *FTa1* activation by the VEG2-FTa1 complex [equation (16) in Materials and Methods] and fitting the resulted model to the complete data set (MC_Cdata_FTa1 model). The new model did not show improved performance as compared to the MC_Cdata model (Figure 4), thus rejecting the hypothesis.

It was shown for soybean (*Glycine max*); another representative of legumes, that activation of flowering initiation under LD conditions involves different regulatory blocks than under SD conditions (Wu et al., 2019). We investigated whether the model performance can be improved if we use the same proposed regulatory scheme for pea but fit the model to the SD and LD data separately (MC_SDdata and MC_LDdata models, respectively). The SD data comprise the SD part from the wild type and data from the *dne-1* mutant, and the LD data include the LD portion of the wild-type data and data from the *late1-2* and *gigas-2* mutants. MC_SDdata and MC_LDdata showed better performance than the MC_Cdata model for the SD and LD growing conditions, respectively (Figure 4). It should be noted that the comparison between these models is not a rigorous test, because the MC_SDdata and MC_LDdata models were fitted to fewer data points than MC_Cdata for the same number of parameters. However, this computational experiment shows that it is possible to reduce the modeling error by narrowing the model to either SD or LD. This suggests that it may be not feasible to use uniform regulatory assumptions under the two growing conditions. The analysis of individual genes reveals that the MC_LDdata model most significantly improves the expression dynamics of *PIM* and *FTc* (both in the wild type, LD), while MC_SDdata improves the expression dynamics of *DET* (wild type, SD, and *dne-1*) and *FTa1* in the apex (*dne-1*; Supplementary Figures 9–13).

### Dynamical Models Based on Neural Networks

The previously described dynamical models were based on the suggested regulation scheme underlying floral initiation in pea (Figure 1B), so studying these models was aimed at answering the question about the quantitative correspondence between this scheme and the expression data. In the next stage of the study, we developed models without prescribing a specific topology of the gene regulatory network, thus trying to answer the question of what regulations can be inferred from the expression data ab initio. Along with changing the question, we also changed the formalism of ODEs to the neural network method to formulate new models, so as not to be dependent on only one modeling method and, thus, increase the robustness of conclusions.

We developed three models (NN, NN_SDdata, and NN_LDdata) based on neural networks, all of which were constructed on the same principles and differed from each other only by the data used for their training. The models were formulated as dynamical regression models in which the apical expression of all genes on the current day was determined by the apical expression of the same genes and the expression of the *FT*-genes from the leaves from the previous day (Podolny et al., 2020). As VEG1 data were present only in LD, we excluded VEG1 from the model for simplicity; we also considered VEG2 as an independent variable. The NN model was trained on the full data (wild-type, *dne-1*, *late1-2*, and *gigas-2*), NN_SDdata on the SD portion of the full data (wild-type, SD, and *dne-1*), and NN_LDdata on the LD portion of the full data (wild-type, LD, *late1-2*, and *gigas-2*). For the NN and NN_SDdata models, we separated data from several days for each condition (daylight and genotype) as the testing dataset, and all data from the *late1-2* mutant were used as the testing dataset for the NN_LDdata model.

**Figure 5:**
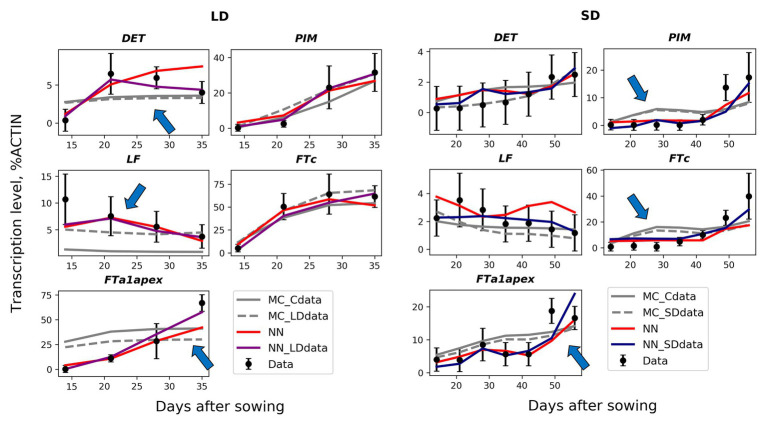
Best solutions in the neural network models (NN, NN_SDdata, and NN_LDdata) in comparison with the models based on the proposed regulation scheme and ordinary differential equations (ODEs; MC_Cdata, MC_SDdata, and MC_LDdata), for the wild type and two growing conditions. The black dots and error ranges are the mean expression and standard deviation, respectively, in the data. The arrows indicate the improvements in expression dynamics achieved in the neural network models.

**Figure 6:**
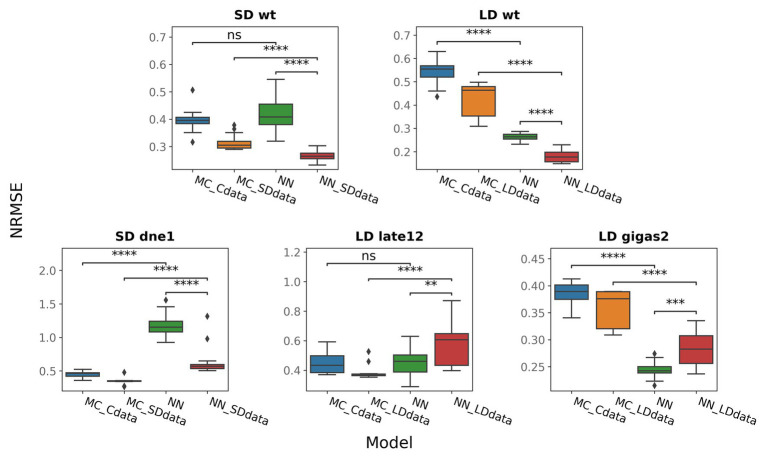
NRMSE for neural network models (NN, NN_SDdata, and NN_LDdata) and models based on ODEs (MC_Cdata, MC_SDdata, and MC_LDdata), for different genotypes and growing conditions. NRMSE was calculated for five genes shown in Figure 5. For *gigas-2*, PIM was excluded from the NRMSE calculation in NN and NN_LDdata as PIM was also excluded from the ODE-based models by construction. The two-tailed Mann–Whitney–Wilcoxon test was applied to check the performance difference between the indicated models; *p*-values: (ns) 0.05 < *p* ≤ 1, (^**^) 0.001 < *p* ≤ 0.01, (^***^) 10^−4^ < *p* ≤ 0.001, (^****^) *p* ≤ 10^−4^.

The solutions in the NN_SDdata and NN_LDdata models show better correspondence to the wild-type data compared to the models based on the proposed regulation scheme (Figures 5–6). There are improvements in expression dynamics of *DET*, *LF*, and *FTa1* in the apex under the LD conditions and of *PIM*, *FTc*, and *FTa1* in the apex under the SD conditions (arrows in Figure 5). The solutions in the NN model is close to NN_SDdata and NN_LDdata but have defects for *FTa1* and *DET* in LD and for *LF* and *FTc* in SD. In contrast to the wild-type data, the neural network models do not show a definite difference with the ODE-based models on the mutant data (Figure 6). NN and NN_LDdata are better for *gigas-2*, while the comparison is in favor of the ODE-based models for the other two mutants. A worse performance of NN_LDdata for *late1-2* can be explained by the fact that the whole data from this mutant were used as a testing set in this model.

**Figure 7:**
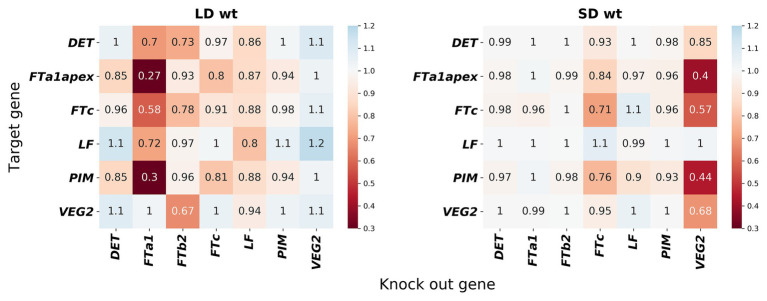
Gene interactions predicted by the neural network models on the wild-type data. The heatmaps show gene knockout simulations in (left) the NN_LDdata and (right) NN_SDdata models; similar results for the NN model and for all three models but on the mutant data are shown in Supplementary Figures 14, 15. Gene knockouts were simulated as described in Materials and Methods. Values below 1 mean activation, above 1 mean repression, and equal to 1 mean no interaction.

In order to understand what interactions were restored in the neural network models, we simulated gene knockouts in the models. In these knockouts, we set a potential regulator protein concentration to zero in the model and calculated how the area under the dynamic expression curve changed for each potential target as the result of such perturbation. We kept the concentrations of all other proteins fixed at their values from the data during this simulation in order to estimate the direct influence of the regulator on the target, excluding possible feedbacks from other genes whose dynamics may also be altered by the perturbation. The resulted gene network topology exhibits some deviations from the proposed regulation scheme from Figure 1B and is qualitatively different in SD and LD (Figure 7). In the proposed regulation scheme, FTa1 is the only activator of the floral meristem identity gene *PIM*, while the neural network models predict FTc as an additional activator both in SD and LD. Other noticeable differences include strong *FTa1* self-activation in LD and *FTc* self-activation in SD.

Concerning differences between LD and SD, the regulatory topology exhibits more activation on the whole in LD compared to SD (Figure 7). Interestingly, VEG2 and FTa1 are predicted to be independent activators. VEG2 is the main activator in SD, with FTa1 almost not influencing other genes. FTa1 serves as the main activator in LD, while VEG2 is either non-active or even shows some repressive potential under this growing condition. Overall, these results show that the improvement in the solution quality demonstrated by the neural network models comes at the price of perturbations to the regulation scheme from Figure 1B.

## Discussion

The classical approach to elucidating functional regulations in a gene network consists in obtaining and qualitatively analyzing the expression patterns of genes involved in the network in various genetic backgrounds. As more data are collected on the genes controlling floral initiation in legumes (Hecht et al., 2011; Sussmilch et al., 2015; Ridge et al., 2017; Cheng et al., 2018), more quantitative approaches are required to infer the interactions in the gene regulatory network underlying this process (Jaeger et al., 2013; Leal Valentim et al., 2015; Gursky et al., 2018). Just as it has successfully been done for *Arabidopsis*, modeling gene networks responsible for the transition to flowering in legumes can be used for testing various hypotheses about the network structure and other properties of the process, in order to better understand the mechanism or to find possible flaws in the current understanding. In this study, we elaborated several models of the core gene network involved in flowering initiation in pea and applied them to the previously obtained expression data in the wild type and in mutants. In order to make our results more robust, we used two different methods to construct models. We showed that both formalisms, ODEs and neural networks, can be utilized to formulate dynamical models suited for the gene expression data used in the study.

Our modeling results indicate that the regulation scheme that was previously proposed by analyzing the expression data qualitatively does not fully correspond to these expression data at the quantitative level. There are two types of evidence in our results for this conclusion. Firstly, the best models implementing the proposed gene regulations (the MC and MC_Cdata models) consistently generated solutions with defects in the expression dynamics of several genes. These defects comprise wrong expression dynamics of *PIM* and *FTc* in SD, *LF* in LD, and inconsistent apical expression of *FTa1*. Moreover, we showed that this picture cannot be fixed by targeted and fine-tuned modifications of the regulation scheme. The rejected alternative interactions include repression of *PIM* by LF, cooperative activation of *PIM* by the VEG2-FTa1 complex, and *FTa1* self-activation. As a floral meristem identity gene, *PIM* is of a special interest. One of the strongest constrains for introducing new potential activators of *PIM* for testing in the model is in the fact that *PIM* expression is almost zero in *FTa1* mutant *gigas-2* (Hecht et al., 2011). Therefore, more complicated regulatory modules have to be devised to provide an additional activation to *PIM*, so that they can be deactivated in the absence of FTa1.

Secondly, gene interactions reconstructed from the data by the neural network-based dynamical models contain new regulations compared to the proposed scheme. It is interesting that one of these new regulations was *FTa1* self-activation, which was rejected at the stage of fine-tuning the proposed scheme with the help of the ODEs-based modeling. This is an example of a hypothesis about a new regulation that does not work when implemented alone but fits in when the regulation acts in concert with other modifications. Another such new regulation is *PIM* activation by FTc. The solution for PIM in the neural network model with this regulation is not zero on the *gigas-2* data but is small enough to stay within error ranges (Supplementary Figure 16), i.e., the activation by FTc is compensated by all other *PIM*’s regulators in the absence of FTa1.

Our modeling results also support the possibility that different regulatory modules are active in SD and LD. The models based on the proposed regulation scheme show the best performance when fitted to the SD and LD data separately. The use of these data in the neural network models lead to qualitatively different regulatory topologies. In SD, VEG2 acts as the main activator, while FTa1 does not play a significant role, and the opposite situation is observed in LD. This possible activating role of VEG2 is in accordance with a previously obtained result showing that the model of floral initiation in *Arabidopsis* is effective under the assumption that FD (VEG2) can activate *AP1* (*PIM*) as a monomer (Leal Valentim et al., 2015). However, it is also possible that this VEG2 and FTa1 decoupling somehow reflects the activating role of the VEG2-FTa1 complex captured by the model differently for different daylight conditions. Another finding about VEG2 concerns cooperative binding in the formation of complexes between VEG2 and FT proteins, which appears to be less favorable than the assumption about binding without constraints. This result can indicate that FTa1, FTb2, and FTc bind VEG2 without essential competition.

Not all regulations predicted by the neural network approach should be considered as real, so that conclusions about those regulations should be made with caution. The inconsistencies observed in the models based on the prescribed regulation scheme most probably mean that some important regulators are missing. A nonlinear response of the gene network to the unknown dynamic expression of these unknown regulators can be encoded in spurious interactions between the genes in the current version of the network. The defects in the model solutions highlight possible genes involved in missing regulations and, thus, can be used to plan further experimental searches.

## Materials And Methods

### Flowering Gene Expression Data

For model calibration, we used previously published dynamic expression data of genes responsible for flowering initiation in pea (cultivar NGB5839; Hecht et al., 2011; Sussmilch et al., 2015). The expression data in the wild type and in mutants were extracted from the published sources using the web-based tool WebPlotDigitizer (Rohatgi, 2018). The data represent the means and SDs of the expression levels of the following genes: *FTa1*, *FTb2*, *FTc*, *DET*, *LF*, *VEG1*, *VEG2*, and *PIM*. The wild type data comprise the expression dynamics from 7 to 35th days after sowing under LD conditions and from 7 to 56th days under SD. Only LD data in the wild type were available for *VEG1*. The mutant data contain the gene expression dynamics from the mutants *dne-1* (mutation in the *DNE1* gene; 7–35 days after sowing under SD), *late1-2* (mutation in *LATE1*; 14–56 days under LD), and *gigas-2* (mutation in *FTa1*; 7–56 days under LD).

### Dynamical Model Based on Differential Equations

We use the same methodology to construct the model as in Gursky et al. (2018). We model the expression of *DET*, *PIM*, *VEG1*, *LF*, *FTc*, and *FTa1* in the apex with the following set of ODEs:

$$\begin{array}{l} \frac{du_{DET}}{dt}=v_{1}\left(\frac{u_{VEG2\;FTb2}}{K_{1}+u_{VEG2\;FTb2}}\right)\\ \;\;\;\;\;\;\;\;\;\;\;\;\;\;\;\;\;\;\;\;\;\;\;\;\;\;\;\;\;\;\;\left(\frac{K_{2}}{K_{2}+u_{VEG1}}\right)-\lambda_{1}u_{DET}\; \end{array}$$ (1)

$$\begin{array}{c} \begin{array}{l} \frac{du_{PIM}}{dt}=v_{2}\left(\frac{u_{VEG2\;FTa1}{}^{n}}{K_{3}{}^{n}+u_{VEG2\;FTa1}{}^{n}}\right)\\ \;\;\;\;\;\;\;\;\;\;\;\;\;\;\;\;\;\;\;\;\;\;\;\;\;\;\;\;\;\left(\frac{K_{4}}{K_{4}+u_{DET}}\right)-\lambda_{2}u_{PIM} \end{array} \end{array}$$ (2)

$$\begin{array}{c} \begin{array}{l} \frac{du_{VEG1}}{dt}=v_{3}\left(\frac{u_{VEG2\;FTa1}}{K_{5}+u_{VEG2\;FTa1}}\right)\left(\frac{K_{6}}{K_{6}+u_{DET}}\right)\\ \;\;\;\;\;\;\;\;\;\;\;\;\;\;\;\;\;\;\;\;\;\;\;\;\;\;\;\;\;\;\;\;\left(\frac{K_{7}}{K_{7}+u_{PIM}}\right)\left(\frac{K_{8}}{K_{8}+u_{LF}}\right)-\lambda_{3}u_{VEG1} \end{array} \end{array}\;$$ (3)

$$\begin{array}{c} \frac{du_{LF}}{dt}=v_{4}\;\frac{K_{9}}{K_{9}+u_{VEG2\;FTb2}}-\lambda_{4}u_{LF}\; \end{array}\;$$ (4)

$$\begin{array}{c} \frac{du_{FTc}}{dt}=v_{5}\left(\begin{array}{l} \frac{u_{VEG2\;FTa1}}{K_{10}+u_{VEG2\;FTa1}}+\\ \frac{u_{VEG2\;FTb2}}{K_{11}+u_{VEG2\;FTb2}} \end{array}\right)-\lambda_{5}u_{FTc} \end{array}\;$$ (5)

$$\begin{array}{c} \frac{du_{FTa1\;apex}}{dt}=v_{6}\frac{u_{VEG2\;FTb2}}{K_{12}+u_{VEG2\;FTb2}}-\lambda_{6}u_{FTa1\;apex} \end{array}$$ (6)

where $u_{i}$ describes protein concentrations. The concentrations of complexes of VEG2 with the FT proteins are denoted as $u_{VEG2\;FTa1}$ in the case of FTa1, and similarly for other FTs. As *FTa1* is expressed both in the leaves and in the apex, the concentration of apically expressed proteins is written as $u_{FTa1\;apex}$. The parameters *v_i_* are the maximal protein synthesis rates, and *K_i_* are the Michaelis–Menten constants, which can be interpreted as equilibrium dissociation constants for regulator-promoter binding in the case of direct transcriptional regulation. The Hill constant *n* is used to account for the potential cooperative binding effect in *PIM* regulation by the VEG2-FTa1 complex; *n* = 1 in all versions of the model except the MM_PIM model, in which *n* was a free parameter. The parameters $\lambda_{i}$ are protein degradation constants. The translation process is not explicitly considered in these equations; we assume that protein concentrations are proportional to the concentrations of corresponding mRNAs.

FTb2 in the apex comprises the protein transported from the leaves, while FTa1 in the apex additionally include the apically expressed fraction. Considering a time delay 𝜏 for the transport process, we write the total apical concentrations $u_{FTa1}$ and $u_{FTb2}$ of FTa1 and FTb2, respectively, as follows:

$$\begin{array}{c} u_{FTa1}\left(t\right)=u_{FTa1\;apex}\left(t\right)+u_{FTa1\;leaf}\left(t-\tau \right)\; \end{array}\;$$ (7)

$$\begin{array}{c} u_{FTb2}\left(t\right)=u_{FTb2\;leaf}\left(t-\tau \right)\; \end{array}\;$$ (8)

where $u_{FTa1\;leaf}$ and $u_{FTb2\;leaf}$ are the concentrations of corresponding proteins expressed in the leaves.

The baseline model considers competitive binding between VEG2 and FTa1, FTb2, and FTc. Under equilibrium competitive binding conditions, the concentrations of VEG2 complexes with the corresponding FT proteins are as follows:

$$\begin{array}{c} u_{VEG2\;FTa1}=\frac{K_{13}u_{FTa1}u_{VEG2}}{1+K_{13}u_{FTa1}+K_{14}u_{FTb2}+K_{15}u_{FTc}}\; \end{array}$$ (9)

$$u_{VEG2\;FTb2}=\frac{K_{14}u_{FTb2}u_{VEG2}}{1+K_{13}u_{FTa1}+K_{14}u_{FTb2}+K_{15}u_{FTc}}\;$$ (10)

$$u_{VEG2\;FTc}=\frac{K_{15}u_{FTc}u_{VEG2}}{1+K_{13}u_{FTa1}+K_{14}u_{FTb2}+K_{15}u_{FTc}}\;$$ (11)

Therefore, the baseline model MM consists of the equations (1)–(11).

### Model Modifications to Test Alternative Hypotheses

The MM_LF model is equivalent to MM but with an additional repression of *PIM* by LF, introduced by adding a repressive term into equation (2) as follows:

$$\begin{array}{c} \begin{array}{l} \frac{du_{PIM}}{dt}=v_{2}\left(\frac{u_{VEG2\;FTa1}{}^{n}}{K_{3}{}^{n}+u_{VEG2\;FTa1}{}^{n}}\right)\\ \;\;\;\;\;\;\;\;\;\;\;\;\;\;\;\;\;\;\;\;\;\;\;\;\;\;\;\;\;\;\;\left(\frac{K_{4}}{K_{4}+u_{DET}}\right)\left(\frac{K_{16}}{K_{16}+u_{LF}}\right)-\lambda_{2}u_{PIM}\; \end{array} \end{array}$$ (12)

The MC model is equivalent to MM but with the binding between VEG2 and FT proteins assumed to be noncompetitive. Under this assumption, the concentrations of complexes are written as follows:

$$\begin{array}{c} u_{VEG2\;FTa1}=u_{FTa1}u_{VEG2}\; \end{array}\;$$ (13)

$$\begin{array}{c} u_{VEG2\;FTb2}=u_{FTb2}u_{VEG2} \end{array}$$ (14)

$$\;\begin{array}{c} u_{VEG2\;FTc}=u_{FTc}u_{VEG2}\; \end{array}\;$$( 15 )

It is not necessary to add free constants of proportionality into (13)–(15), since they can be effectively scaled into free *K_i_* already present in equations (1)–(6).

The MC_PIM model is equivalent to MC but leaves the Hill parameter *n* free in equation (2). This value, together with values of all other parameters, is found by parameter optimization. A value *n* larger than one would suggest the cooperative binding of the VEG2-FTa1 complex to the promoter of *PIM*.

The models described above were fitted to the wild type data. The MC_Cdata model is the model MC in which the values of free parameters were found by fitting to combined data, which join the wild-type data and data from *dne-1*, *late1–2*, and *gigas-2* mutants. The MC_Cdata_FTa1 model is equivalent to MC_Cdata but with added *FTa1* self-activation in the apex, which was introduced by changing equation (6) to the following one:

$$\begin{array}{c} \frac{du_{FTa1\;apex}}{dt}=v_{6}\left(\begin{array}{l} \frac{u_{VEG2\;FTb2}}{K_{12}+u_{VEG2\;FTb2}}+\\ \frac{u_{VEG2\;FTa1}}{K_{17}+u_{VEG2\;FTa1}} \end{array}\right)-\lambda_{6}u_{FTa1\;apex} \end{array}\;$$ (16)

The MC_SDdata model is the MC model in which free parameters were found by fitting to the combined SD data, consisting of the SD part of the wild type data and *dne-1* mutant data. Similarly, MC_LDdata is the MC model fitted to the combined LD data, consisting of the LD part of the wild type data together with *late1-2* and *gigas-2* mutant data. Supplementary Table 1 summarizes all the models investigated in the study with their main characteristics.

Numerical solutions of the model equations were obtained using the *ode23s* solver in Octave. The concentrations of all regulators on the right-hand side of the equations were replaced by data interpolated in time. The initial conditions for all proteins were set to the data values at the first day.

### Parameter Optimization

The parameter values were found by minimizing the following weighted residual sum of squares (wRSS):

$$\begin{array}{c} wRSS=\overset{N}{\underset{g=1}{\sum }}\overset{T}{\underset{k=1}{\sum }}\frac{{\left(u_{g}\left(t_{k}\right)-u_{g}^{dat}\left(t_{k}\right)\right)}^{2}}{{\left(u_{g,\max}-u_{g,\min}\right)}^{2}} \end{array}$$ (17)

where the difference between the model solution $u_{g}$ and the data $u_{g}^{dat}$for gene *g* is summed over all genes and times for which the data is available; $u_{g,\max}$ and $u_{g,\min}$ are the maximum and minimum concentrations in the data for gene *g*. Since VEG1 data was absent in SD, the numerical solution for this protein was calculated in the model but did not participate in the cost function (17). The data portion (wild type, mutant, SD, and LD) used in equation (17) depended on a model, as described above. This cost function was minimized using the DEEP software, which implements an entirely parallelized version of the differential evolution optimization method (Kozlov et al., 2016).

To reduce the number of free parameters in the models, we set *λ_i_* = 0.199 for all proteins based on an experimental estimate of 3.49 days for the protein half-life in *Arabidopsis* grown at 20°C (Ishihara et al., 2015). To further reduce the possibility for overfitting, we applied an ensemble approach (Samee et al., 2015; Gursky et al., 2018). The optimization for each model was performed 20 times, and the judgment about the model performance was made by analyzing the resulted distribution of the wRSS values.

We compared the models using the normalized root-mean-square deviation:

$$\begin{array}{c} NRMSE=\sqrt{\frac{1}{NT}\overset{N}{\underset{g=1}{\sum }}\overset{T}{\underset{k=1}{\sum }}\frac{{\left(u_{g}\left(t_{k}\right)-u_{g}^{dat}\left(t_{k}\right)\right)}^{2}}{{\left(u_{g,\max}-u_{g,\min}\right)}^{2}}} \end{array}\;$$ (18)

The Mann–Whitney–Wilcoxon test was used to compare the normalized root-mean-square error (NRMSE) distributions resulted from the parameter optimization in the models.

### Neural Network Models

The neural network models were constructed as described in full details elsewhere (Podolny et al., 2020). The data set was expanded to 1,000 gene expression values per time point by sampling from normal distributions with the mean and variance taken from the initial data. The expanded data set was used for training and testing the models. The models were constructed as dynamical regression models in which the apical expression levels of six target genes (*DET*, *PIM*, *FTc*, *FTa1*, *LF*, and *VEG2*) on the current day was determined by the apical expression levels of seven genes (*DET*, *PIM*, *FTc*, *FTa1*, *LF*, *VEG2*, and *FTb2*) taken from the expanded data on the previous day.

The models were trained using the multilayer-perceptron regressor “MLPRegressor” of the Scikit-learn package (Pedregosa et al., 2011), with $f\;\left(x\right)={\left(1+\exp\left(-x\right)\right)}^{-1}$ as the activation function and the Adam stochastic method as the parameter optimization method (Kingma and Ba, 2015). The network architecture was chosen by training the models with different topologies and picking up the best one. Each model was trained 20 times using 5-fold cross-validation, and the ensemble approach was applied for the performance analysis, as described above.

The NN model is the neural network model trained on the combined data (wild type and all mutant conditions). The NN_SDdata model was trained on the SD data (SD wild type data and mutant *dne-1* data). For these two models, the testing sample was constructed by taking data values from the last day of each separate condition. The NN_LDdata model was trained on the portion of the LD data that included the LD wild-type data and the *gigas-2* mutant data, while data from the *late1–2* mutant served as a testing sample for this model. Supplementary Table 2 summarizes the neural models with their main characteristics.

### Simulating Knockouts in Neural Network Models

In order to find out which interactions between genes are restored in the neural network models, a gene knockout analysis was performed. The models were tested on the wild type data in which the expression of one regulator gene was set to zero. Then the AUC of a target gene expression dynamics was calculated in this simulation (*S*_knock_out_) and in the non-perturbed case (*S*_wt_). The ratio of these quantities provides information on the influence type that the regulator directly exerts on the target, as follows:

$$\frac{S_{\mathrm{knock}\_\mathrm{out}}}{S_{\mathrm{wt}}}>1\to \mathrm{repressor},\frac{S_{\mathrm{knock}\_\mathrm{out}}}{S_{\mathrm{wt}}}<1\to \mathrm{activator}$$

## Data Availability Statement

Publicly available datasets were analyzed in this study. This data can be found here: the source code of the models used in the study and analyzed expression data extracted from the published sources can be found in the Zenodo repository (doi: 10.5281/zenodo.4059688; https://zenodo.org/record/4059688#.X8-ho2QzZpQ). The source code of the DEEP software used for parameter optimization in our study can be found in the Gitlab repository (https://gitlab.com/mackoel/deepmethod/-/tree/master).

## Author Contributions

MS and VG conceived and coordinated the study. PP conducted the computational experiments and wrote the first draft of the manuscript. PP, MS, and VG analyzed the results. All authors contributed to the article and approved the submitted version.

### Conflict of Interest

The authors declare that the research was conducted in the absence of any commercial or financial relationships that could be construed as a potential conflict of interest.
