# Supplementary material for: Dynamical Modeling of the Core Gene Network Controlling Transition to Flowering in Pisum sativum
Source: Front Genet. 2021 Mar 11;12:614711. doi: 10.3389/fgene.2021.614711 (PMC7990781; doi:10.3389/fgene.2021.614711)
Supplement: Supplementary file 1 [file Data_Sheet_1.pdf]

Supplementary Material

1 Supplementary Figures and Tables

Supplementary Table 1. Basic characteristics of ODE-based models.

Model	Short name	Data	Number of experimental points	Number of parameters to be optimized
**Michaelis–Menten, competitive binding VEG2 c FTa1, FTb2, FTc**	MM	SD, LD	78	22 free parameters (6 v_i, 15 K_i, τ)
**Michaelis-Menten, competitive binding VEG2 c FTa1, FTb2, FTc, LF -| PIM**	MM_LF	SD, LD	78	23 free parameters (6 v_i, 16 K_i, τ)
**Michaelis-Menten, uncompetitive binding VEG2 c FTa1, FTb2, FTc**	MC	SD, LD	78	19 free parameters (6 v_i, 12 K_i, τ)
**Michaelis-Menten effect of cooperative binding upon activation PIM**	MC_PIM	SD, LD	78	21 free parameters (6 v_i, 2 n_{pim}, 12 K_i, τ)
**Michaelis-Menten combined data**	MC_Cdata	SD, LD, *gigas-2*, *late1-2*, *dne-1*	167	19 free parameters (6 v_i, 12 K_i, τ)
**Michaelis-Menten combined data FTa1→PIM**	MC_Cdata_FTa1	SD, LD, *gigas-2*, *late1-2*, *dne-1*	167	20 free parameters (6 v_i, 13 K_i, τ)
**Michaelis-Menten LD data**	MC_LDdata	LD, *gigas-2*, *late1-2*	89	19 free parameters (6 v_i, 12 K_i, τ)
**Michaelis-Menten SD data**	MC_SDdata	SD, *dne-1*	78	19 free parameters (6 v_i, 12 K_i, τ)

Supplementary Table 2. Basic characteristics of neural networks models.

Model	Train set	Test set	Best configuration of neural network
NN_SDdata	SD, *dne-1*	Last day from each condition	1 hidden layer with 4 neurons
NN_LDdata	LD, *gigas-2*	late12	1 hidden layer with 5 neurons
NN	SD, LD, *dne-1*, *gigas-2*, *late1-2*	Last day from each condition	1 hidden layer with 7 neurons

Supplementary Table 3. The two-sided Mann–Whitney–Wilcoxon test for models MM, MM_LF, MC, and MC_PIM for mutants *dne-1*, *late1-2*, and *gigas-2*.

	**dne-1**	**late1-2**	**gigas2**
**MM vs MM_LF**	P_val=4.928e-01<br>U_stat=2.400e+02	P_val=1.000e+00<br>U_stat=1.690e+02	P_val=4.282e-03<br>U_stat=7.600e+01
**MM vs MC**	P_val=1.296e-02<br>U_stat=3.060e+02	P_val=2.273e-05<br>U_stat=3.660e+02	P_val=1.092e-02<br>U_stat=3.080e+02
**MC vs MC_PIM**	P_val=8.832e-02<br>U_stat=1.190e+02	P_val=1.250e-04<br>U_stat=4.800e+01	P_val=3.596e-01<br>U_stat=1.420e+02

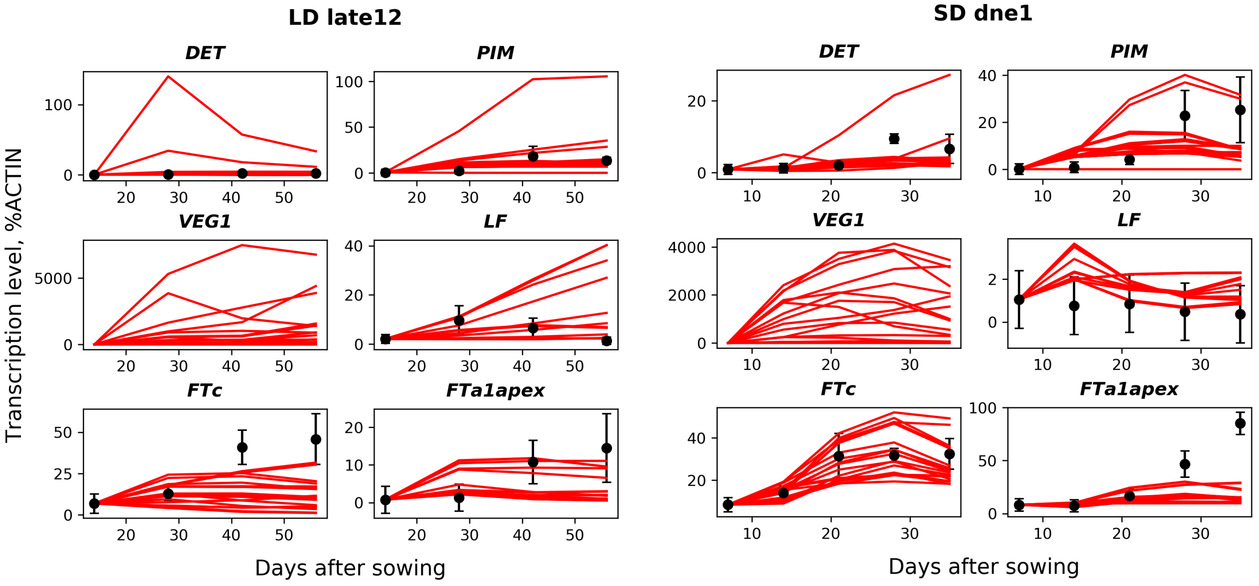

Supplementary Figure 1. Solutions in the MM model for mutant genotypes *late1-2* (left panels) and *dne-1* (right panels). The model solutions (red curves) corresponding to all parameter sets found by optimization are shown for six genes. The black dots and error ranges are the mean expression and standard deviation in data. Data for VEG1 is absent.

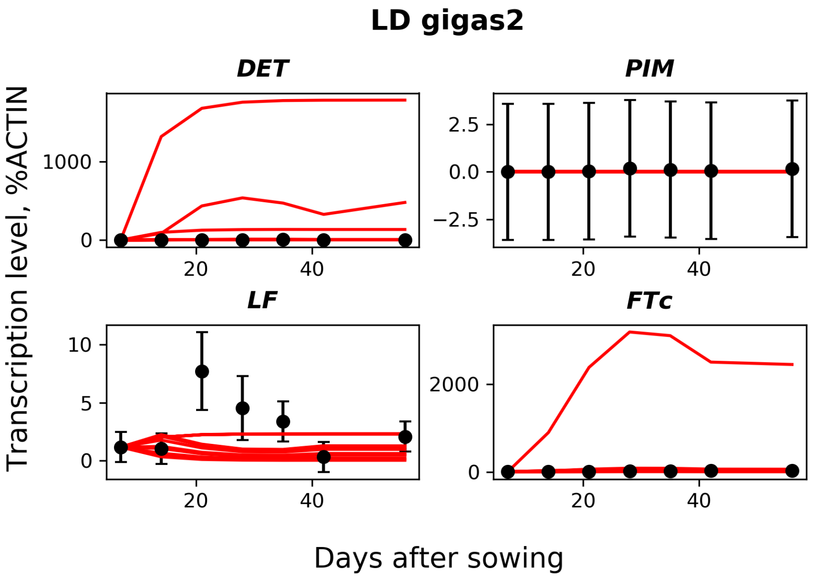

Supplementary Figure 2. Solutions in the MM model for the mutant genotype *gigas-2*. The model solutions (red curves) corresponding to all parameter sets found by optimization are shown. The black dots and error ranges are the mean expression and standard deviation in data. FTa1 is absent in this mutant and is set to zero in the model equations. This leads to zero VEG1 in the model solutions (not shown in the figure since the data for VEG1 is also absent).

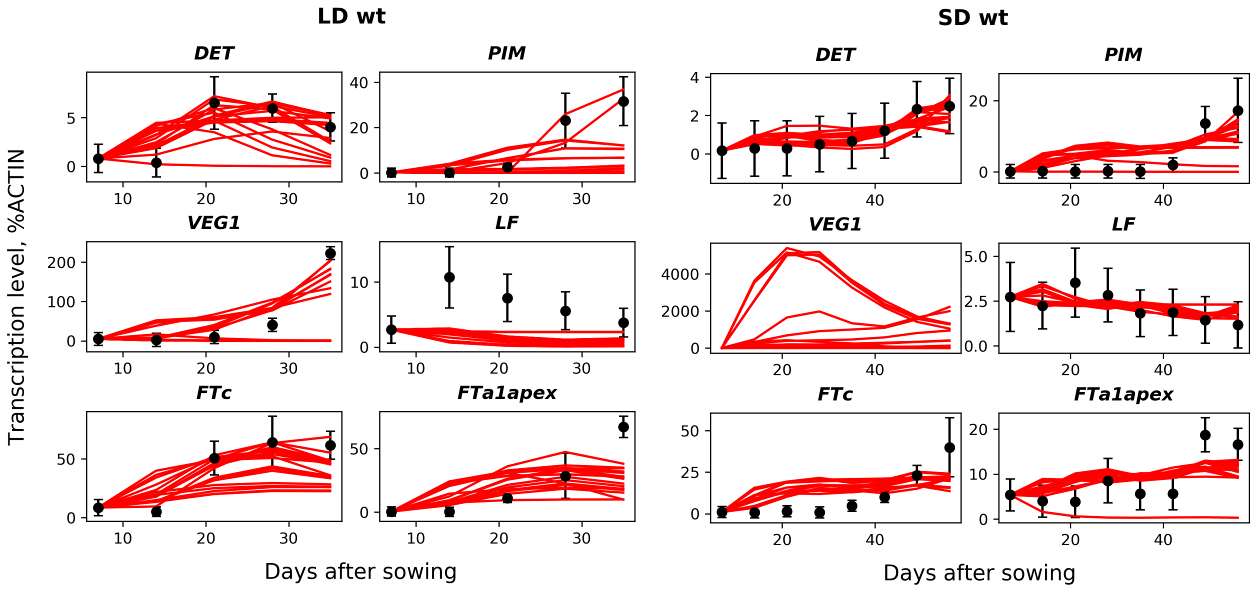

Supplementary Figure 3. Solutions in the MM_LF model on the wild-type data. The model solutions (red curves) corresponding to all parameter sets found by optimization are shown for six flowering time genes and for the short day (SD, right panels) and long day (LD, left panels) conditions. The black dots and error ranges are the mean expression and standard deviation in data.

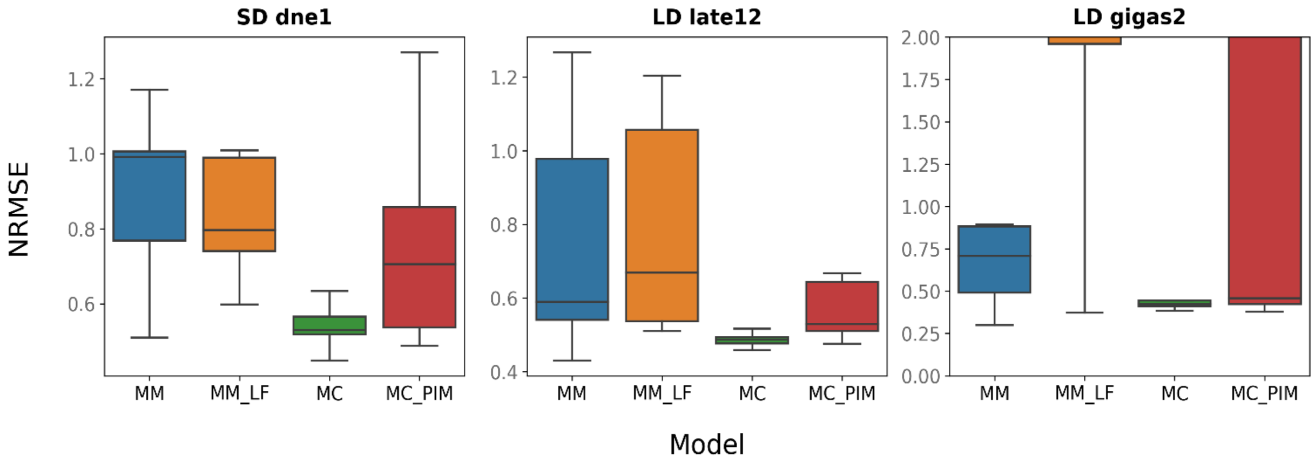

Supplementary Figure 4. Normalized root-mean-square errors (NRMSE) calculated on the mutant data and solutions in models trained on the wild-type data (models MM, MM_LF, MC, and MC_PIM). The boxplot for each model and mutant represents NRMSE values for all parameter sets found by parameter optimization.

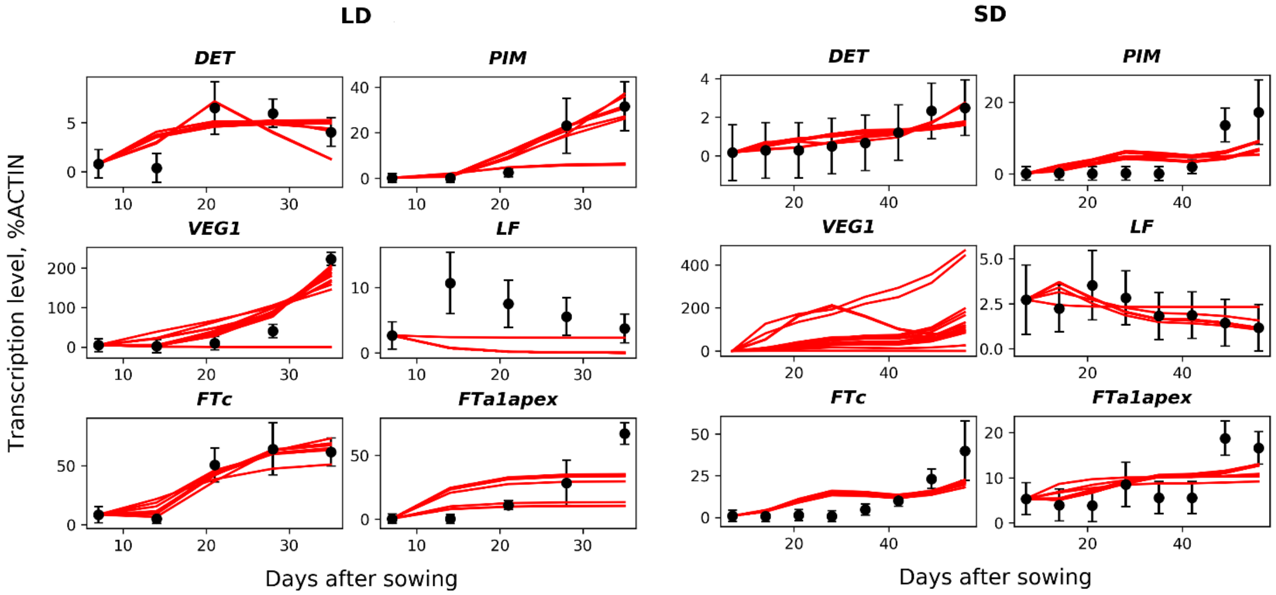

Supplementary Figure 5. Solutions in the MC model on the wild-type data. The model solutions (red curves) corresponding to all parameter sets found by optimization are shown for six flowering time genes and for the short day (SD, right panels) and long day (LD, left panels) conditions. The black dots and error ranges are the mean expression and standard deviation in data.

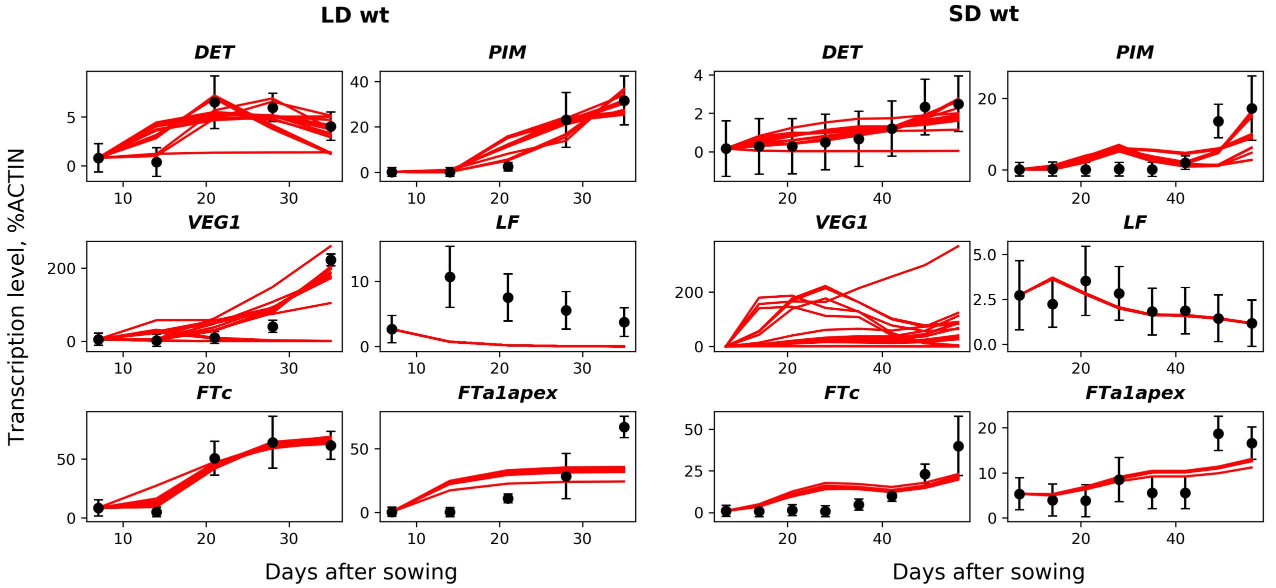

Supplementary Figure 6. Solutions in the MC_PIM model on the wild-type data. The model solutions (red curves) corresponding to all parameter sets found by optimization are shown for six flowering time genes and for the short day (SD, right panels) and long day (LD, left panels) conditions. The black dots and error ranges are the mean expression and standard deviation in data.

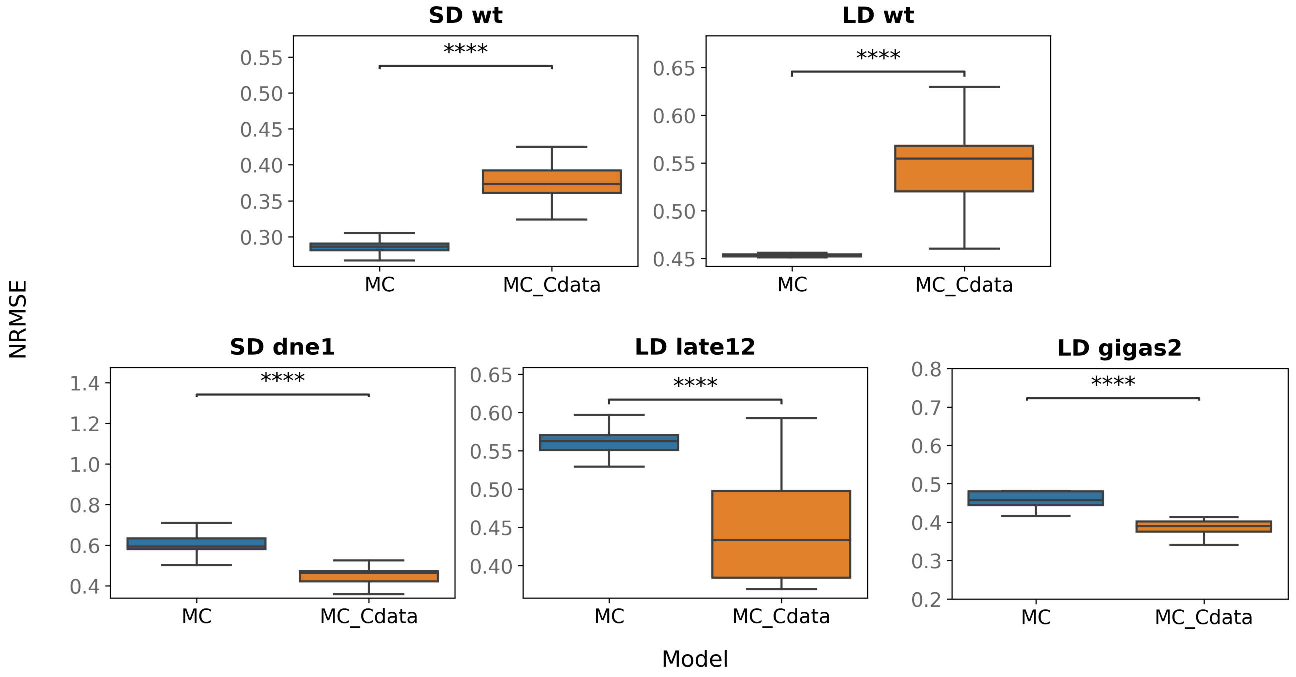

Supplementary Figure 7. Normalized root-mean-square errors (NRMSE) calculated on the wild-type or mutant data and solutions in the MC and MC_Cdata models. The boxplot for each model and data type represents NRMSE values for all parameter sets found by parameter optimization. The Mann–Whitney–Wilcoxon test was applied to check the difference between the two models; *p*-values: (ns) $0.05 < p \leq 1$, (****) $p \leq 10^{-4}$.

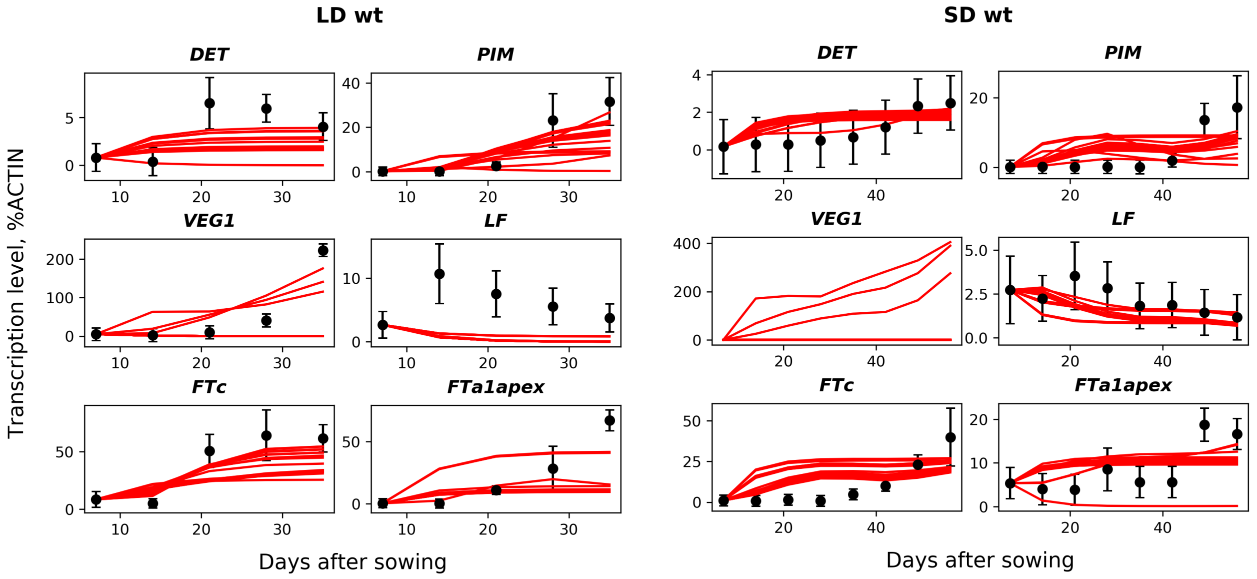

Supplementary Figure 8. Solutions in the MC_Cdata model on the wild-type data. The model solutions (red curves) corresponding to all parameter sets found by optimization are shown for six flowering time genes and for the short day (SD, right panels) and long day (LD, left panels) conditions. The black dots and error ranges are the mean expression and standard deviation in data.

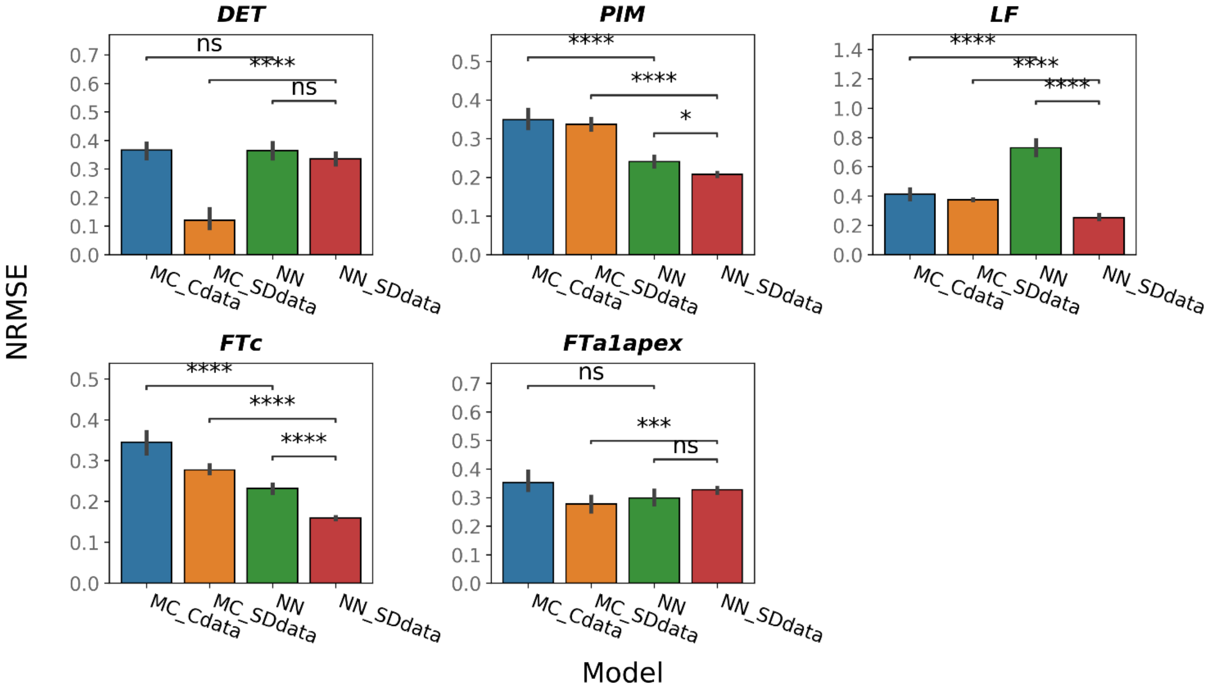

Supplementary Figure 9. Normalized root-mean-square error (NRMSE) in four models (MC_Cdata, MC_SDdata, NN, and NN_SDdata) on the wild-type data under SD conditions. *p*-values: (ns) $0.05 < p \leq 1$, (*) $0.01 < p \leq 0.05$, (***) $10^{-4} < p \leq 0.001$, (****) $p \leq 10^{-4}$.

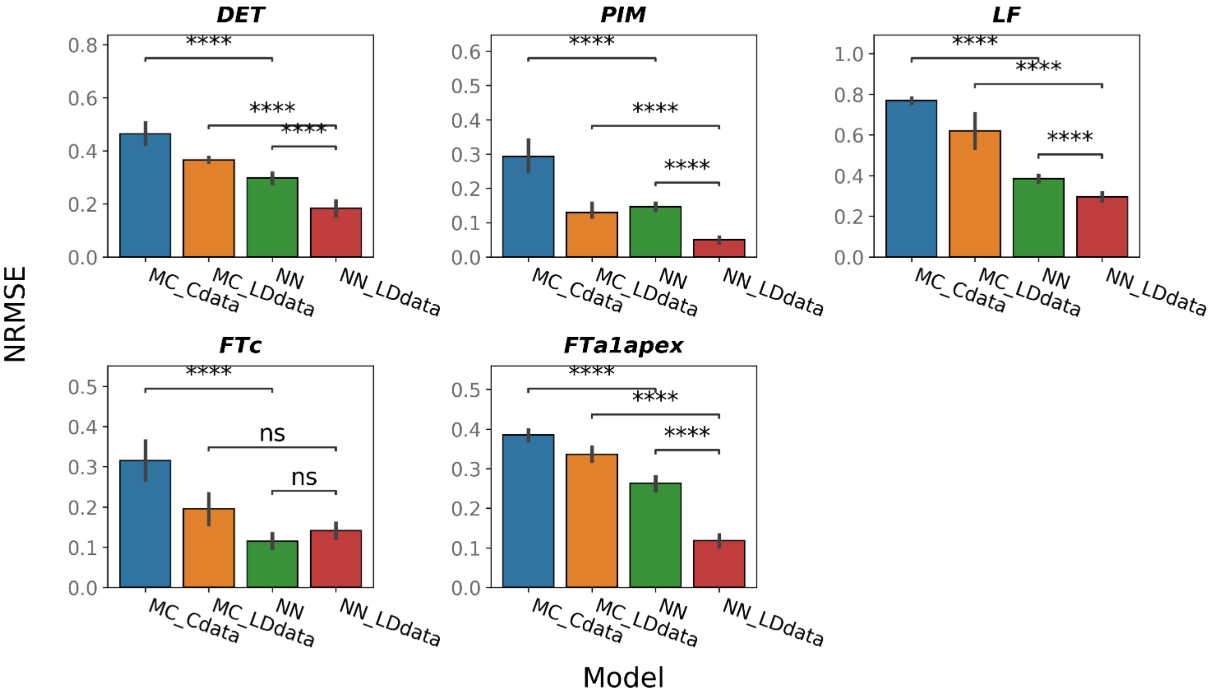

Supplementary Figure 10. Normalized root-mean-square error (NRMSE) in four models (MC_Cdata, MC_LDdata, NN, and NN_LDdata) on the wild-type data under LD conditions. *p*-values: (ns) $0.05 < p \leq 1$, (****) $p \leq 10^{-4}$.

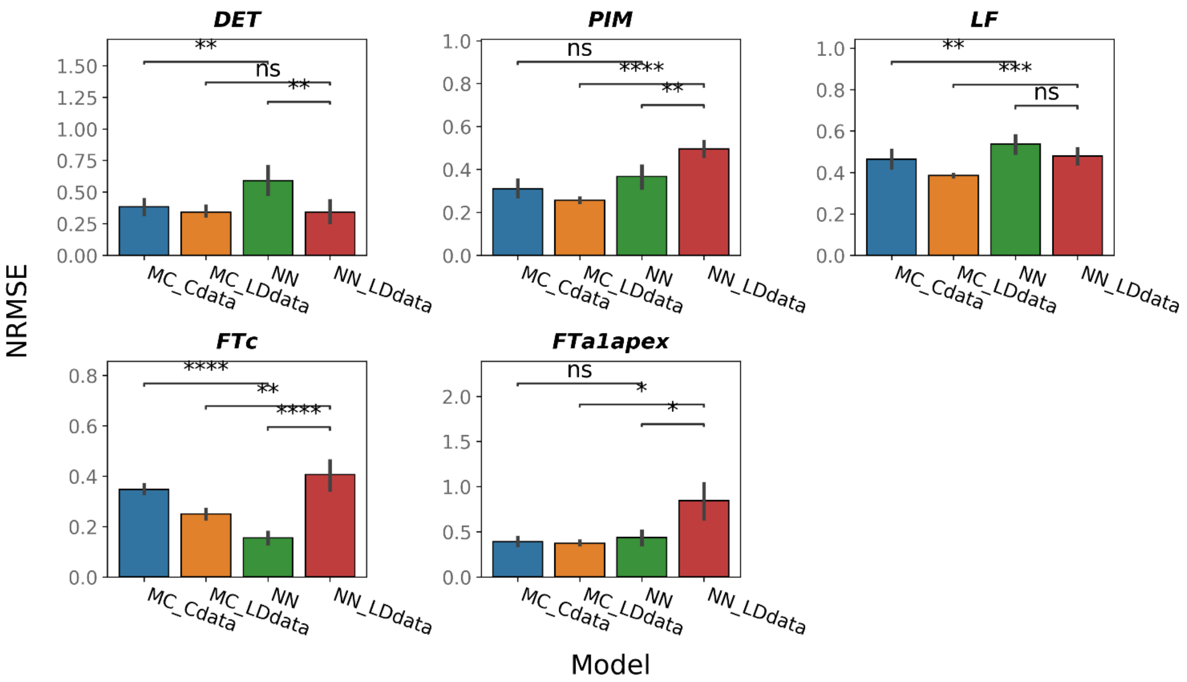

Supplementary Figure 11. Normalized root-mean-square error (NRMSE) in four models (MC_Cdata, MC_LDdata, NN, and NN_LDdata) on data from the *late1-2* mutant under LD conditions. *p*-values: (ns) $0.05 < p \leq 1$, (*) $0.01 < p \leq 0.05$, (***) $10^{-4} < p \leq 0.001$, (****) $p \leq 10^{-4}$.

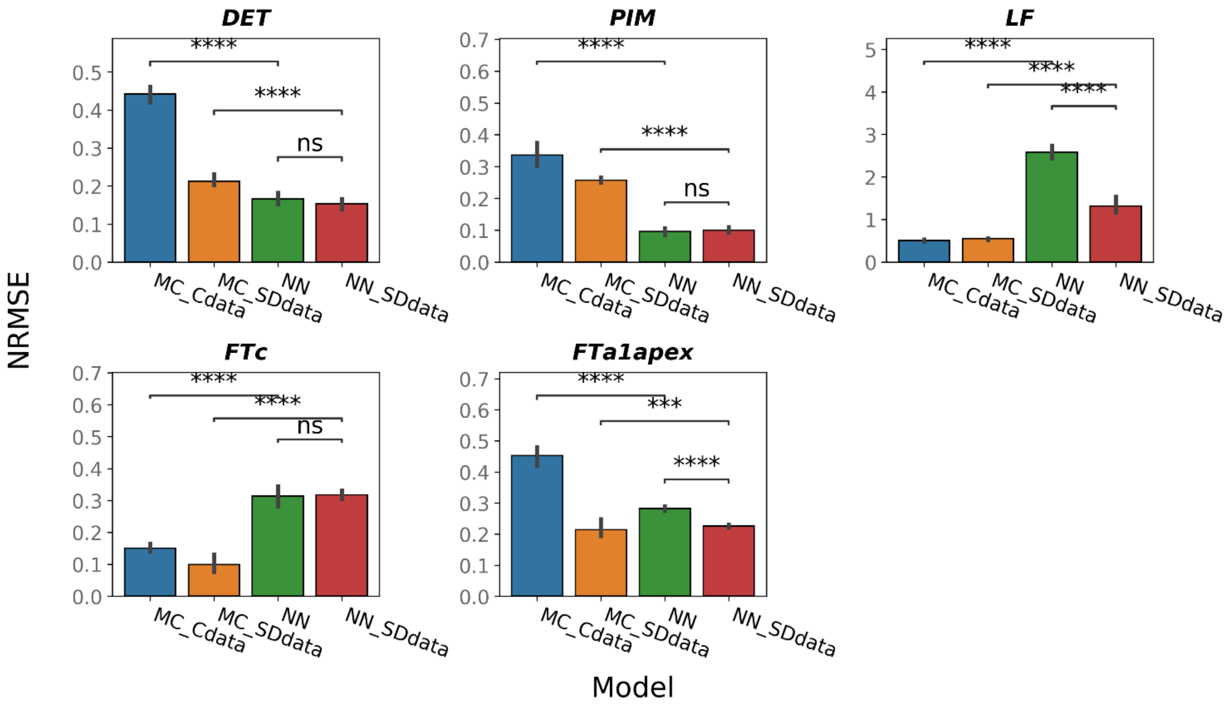

Supplementary Figure 12. Normalized root-mean-square error (NRMSE) in four models (MC_Cdata, MC_SDdata, NN, and NN_SDdata) on data from the *dne-1* mutant under SD conditions. *p*-values: (ns) $0.05 < p \leq 1$, (***) $10^{-4} < p \leq 0.001$, (****) $p \leq 10^{-4}$.

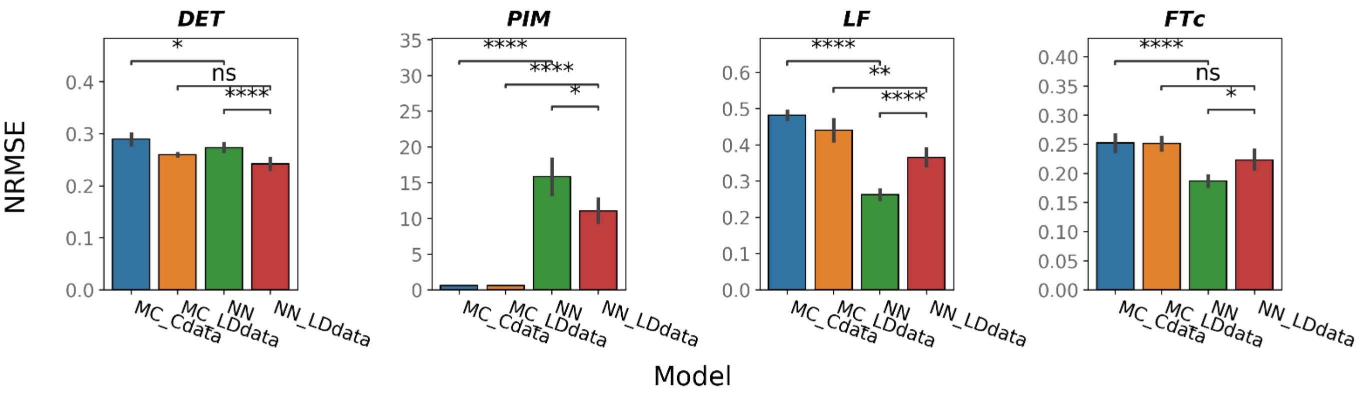

Supplementary Figure 13. Normalized root-mean-square error (NRMSE) in four models (MC_Cdata, MC_LDdata, NN, and NN_LDdata) on data from the *gigas-2* mutant under LD conditions. p-values: (ns) $0.05 < p \leq 1$, (*) $0.01 < p \leq 0.005$, (****) $p \leq 10^{-4}$.

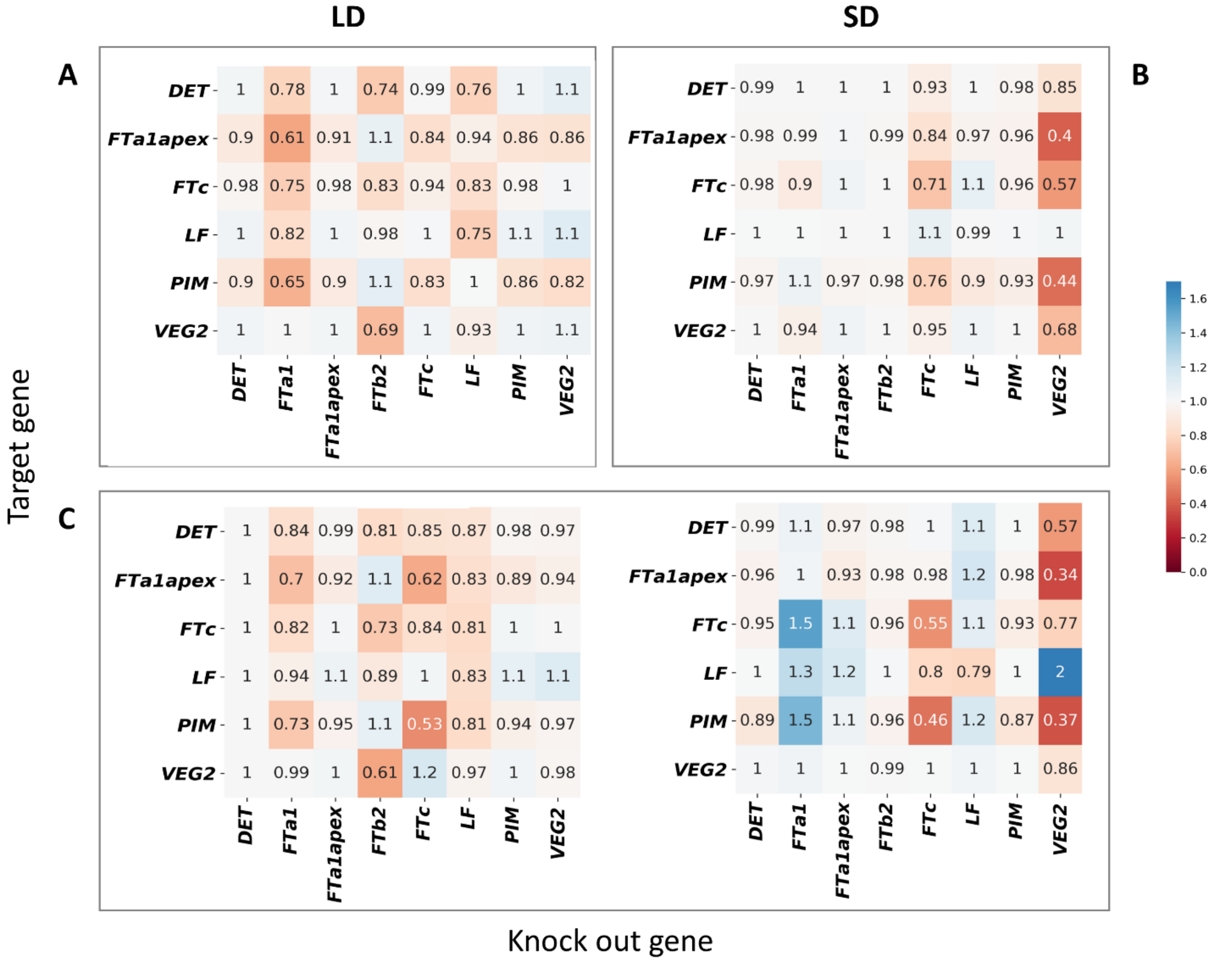

Supplementary Figure 14. (A) Heatmap of gene knockout simulations in the NN_LDdata model on the wild-type data. **(B)** Same as **(A)** but for the NN_SDdata model. **(C)** Same as **(A)** but for the NN model.

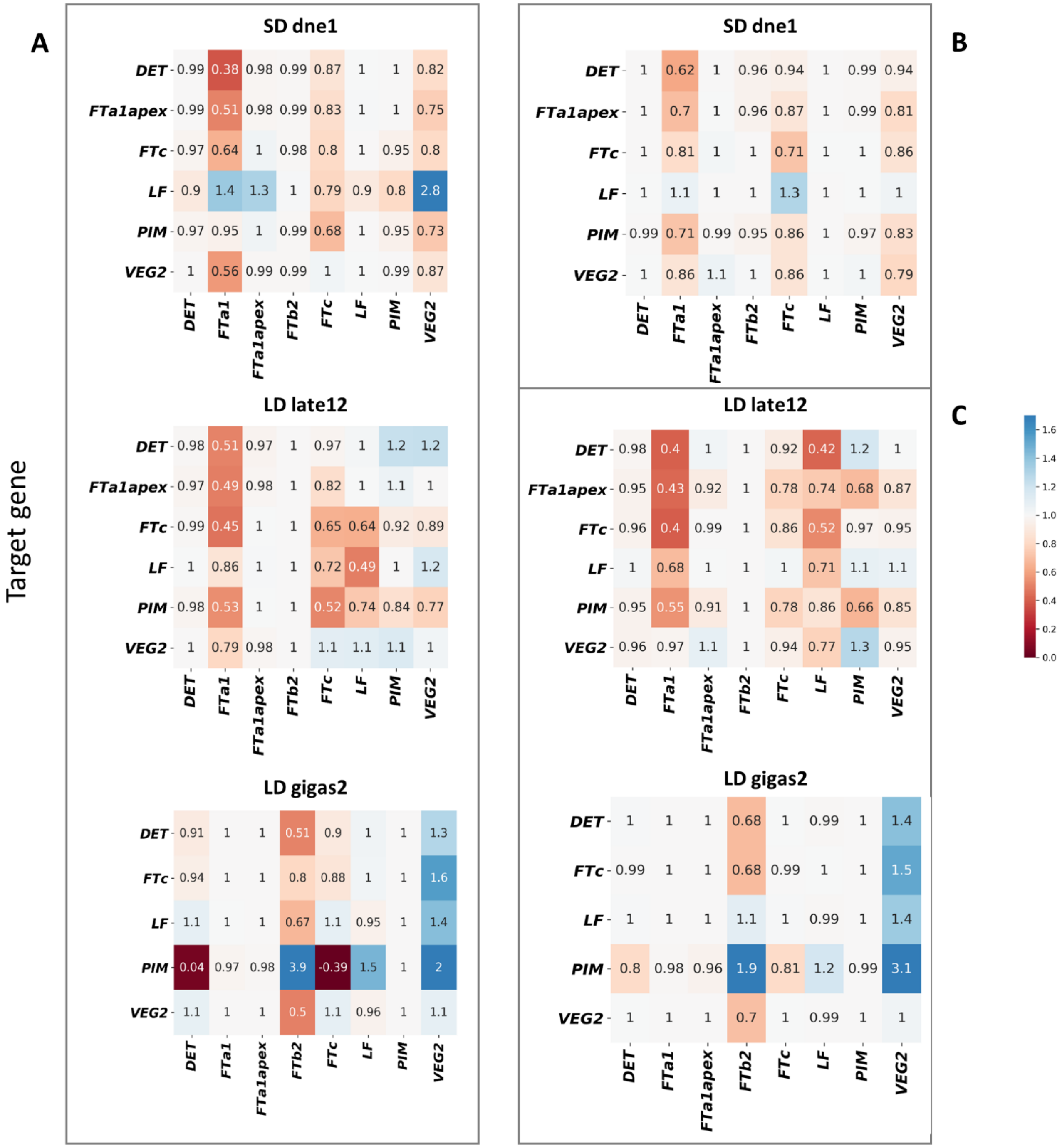

Supplementary Figure 15. (A) Heatmap of gene knockout simulations in the NN model on the mutant data. **(B)** Same as **(A)** but for the NN_SDdata model. **(C)** Same as **(A)** but for the NN_LDdata model.

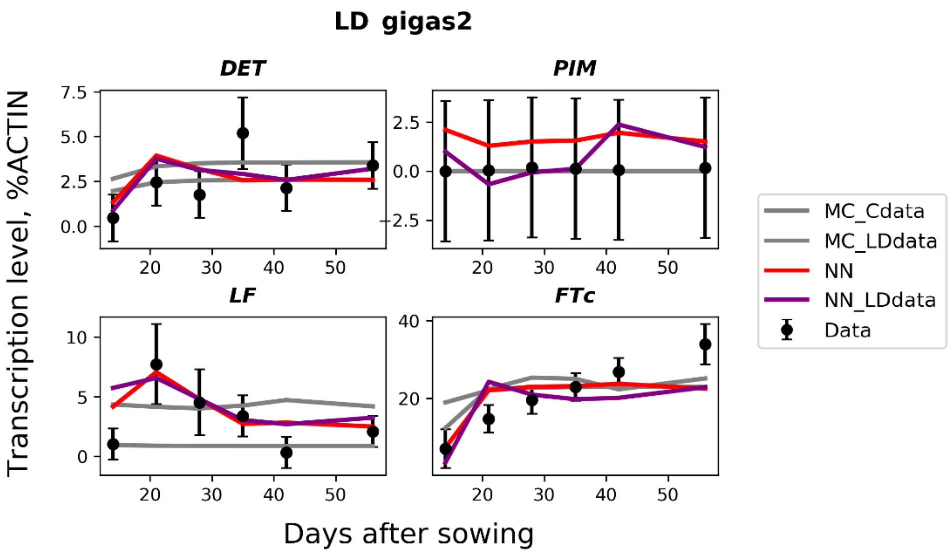

Supplementary Figure 16. Expression dynamics in the neural network models for the *gigas-2* mutant. The model solutions (red and purple curves) are shown for four flowering time genes. The grey curves are solutions in the ODE-based models MC_Cdata and MC_LDdata.
